# A New Approach: Determining *cyt b* G143A Allele Frequency in *Zymoseptoria tritici* by Digital Droplet PCR

**DOI:** 10.3390/biology11020240

**Published:** 2022-02-04

**Authors:** Greta Battistini, Katia Gazzetti, Marina Collina

**Affiliations:** 1 Department of Agricultural and Food Sciences, University of Bologna, Viale G. Fanin 42, 40127 Bologna, Italy; greta.battistini@alice.it (G.B.); k.gazzetti@gmail.com (K.G.); 2 Research Centre for Genomics and Bioinformatics, Council for Agricultural Research and Economics, Via San Protaso 302, 29017 Fiorenzuola d’Arda, Italy

**Keywords:** ddPCR, mutation detection, fungicide resistance, QoIs, cytochrome bc1, Septoria Tritici Blotch

## Abstract

**Simple Summary:**

Droplet digital polymerase chain reaction (ddPCR) is an innovative technique for quantifying a target DNA in a diluted target sample, based on the partition of PCR reaction in a large number of sub-reactions (droplets). In recent years there has been an increase in digital PCR (dPCR) utilization in several different fields, and the application of ddPCR in fungicide resistance studies is very recent. *Zymoseptoria tritici* is the causal agent of Septoria Tritici Blotch (STB), one of the most devasting foliar diseases of wheat grown in temperate climates. STB control relies mostly on fungicide applications and mutations conferring fungicides resistances are concerning phenomena. G143A substitution in fungal cytochrome bc1 confers resistance toward Quinone outside Inhibitor (QoIs) fungicides. In Italy, QoIs are currently sprayed in STB control programs. To the best of our knowledge, we have developed the first ddPCR assay for G143 and A143 alleles detection in samples of gDNA from *Z. tritici* monocondial cultures. We have also investigated G143 and A143 alleles frequency in Italian *Z. tritici* populations representative of different fungicide management strategies. The detection of very low G143A substitution percentages in *Z. tritici* populations is essential for monitoring the emergence of QoIs resistance in field and effectively control STB.

**Abstract:**

*Z. tritici* first appeared in Italy later than in northern-central European countries. QoIs fungicides currently play a role in STB control, used in combination with Demethylation Inhibitors (DMIs) or Succinate dehydrogenase Inhibitors (SDHIs). In this study, we set up a fast, sensitive, and accurate ddPCR protocol in order to investigate the presence and frequency of G143A substitution, causing a reduction in strobilurins’ efficacy in *Z. tritici*. The best PCR conditions for the clear separation of positive and negative droplets were identified. The lowest wild-type and resistant alleles frequencies were accurately determined on samples consisting of mixed DNAs from monoconidial cultures of *Z. tritici* and were expressed as fractional abundance. The protocol was tested by determining the copy number and frequency of alleles on gDNA purified in three Italian *Z. tritici* field populations representative of different fungicide management strategies. For the first time, the determination of allele concentration and the frequency of a mutation involved in *Z. tritici* fungicide resistance was carried out by employing digital PCR. This new approach provides a diagnostic tool that is rapid and able to detect very low G143A substitution percentages, which is very useful for fungicide resistance detection at early stages, thus, informing field management strategies for contrasting STB disease.

## 1. Introduction

Wheat (*Triticum* genus) is an important crop in northern-central Europe but also in Mediterranean countries, such as Italy, especially *Triticum durum* cultivars. Septoria Tritici Blotch (STB), caused by *Zymoseptoria tritici*, can produce heavy yield losses every year when not properly controlled. In Italy the severity of STB infection, on durum wheat varieties, showed an exponential growth trend in the years from 2007 to 2010, reaching a peak in 2013 and a further increase in 2016 [1]. In the plains area of Bologna (Emilia-Romagna, a geo-political region in northern Italy), the infection caused yield losses of up to 30% in years with high inoculum pressure [2]. Genetic resistance against STB is absent or only partial and, consequently, disease control relies mostly on fungicide applications. Demethylation Inhibitors (DMIs), Quinone outside Inhibitors (QoIs), and Succinate dehydrogenase Inhibitors (SDHIs) have been introduced and multisite fungicides are routinely used to control *Z. tritici* [3].

QoIs inhibit mitochondrial respiration by binding to the Qo binding site of the cytochrome bc1 (ubiquinol oxidase, *cyt b*) enzyme complex, causing an electron-transfer block and ATP synthesis [4], and are classified by the Fungicide Resistance Action Committee (FRAC) as fungicides with a high risk of developing resistance [5]. It has been demonstrated that efficacy reduction is caused by the replacement of glycine with alanine at position 143 (G143A) belonging to the *cyt b* Qo site. The first case of resistance on *Z. tritici* was detected in 2001 in the United Kingdom [6], and since then resistance has been identified in several parts of Europe [7,8], starting in 2002, with rapid spreading in North America [9], New Zealand [10], and the Baltic region [11].

In northern-central Europe, resistance to strobilurin fungicides in STB is widespread [11], while in Italy QoIs currently still play a role in STB control when used in combination with DMIs or SDHIs [12,13]. Several factors, such as the low number of fungicide applications, the generally low disease pressure due to less humidity and a shorter wheat vegetative cycle, and the more recent introduction of the pathogen could explain the difference between Italy and other important European wheat producing areas. FRAC reported an Italian G143A strain as early as 2010, and average moderate levels of QoI resistance with high variability were recorded for Italian populations of *Z. tritici* during 2015 and 2017. Even though FRAC classified the status of Italy at the end of the 2019 season as a country with a moderate level of *Z. tritici* resistance to strobilurins with high variability, no broad monitoring data are currently published on fungicide sensitivity. Our laboratory conducted a preliminary monitoring test on the sensitivity of *Z. tritici* strains to QoIs, in order to collect first data about the Italian scenario [14]. The molecular results obtained by Sanger sequencing performed on those samples showed the presence of G143A substitution in some cases [15] and raised the need for a deeper investigation.

The term digital PCR was first used in 1999 [16] and can be based on droplet, microwell, channel, or printing dispersion methods [17]. The very sensitive ddPCR technique is based on the partition of reaction in a large number of sub-reactions (droplets) and enables the quantifying of target DNA in highly diluted target samples. By utilizing fluorescent probes, it is possible to classify the sub-reactions as positive (containing target DNA) or negative (without target DNA). Through Poisson statistics, it is then possible to calculate the original number of DNA target copies (copies µL^−1^) in the starting materials. This model defines M as the average number of target copies per partition, and it is determined with the following equation: M = −ln(1 − P/R), where P is the number of positive partitions and R is the total number of partitions detected [18]. In optimal run conditions, one band should be visible at a low amplitude, representing the negative droplets, and a second band at a high amplitude, representing the positive droplets. It is also possible to perform a multiplex assay using different fluorescent dyes (FAM, HEX/VIC).

In recent years, there has been an increase in ddPCR utilization in several different fields. Most applications have been performed in the medical field, such as quantitative detection of cell-free circulating circRNAs [19]. Even though ddPCR systems are currently used less extensively in the agricultural sector, to date the ddPCR technique has already been applied in experimental evolution studies [20], to quantify GMO organisms [21,22,23], to determine the precise limit of detection (LOD) of the two postharvest biological control agents *Aureobasidium pullulans* L1 and L8 [24], and to achieve the early detection and accurate quantification of *Potato virus Y* strains [25], as well as phytoplasma in plants and insects [26]. The application of digital PCR in fungicide resistance studies is new. An assay was developed in 2018 for the detection and quantification of mutations in the Cyp51 gene of *Blumeria graminis* f. sp. *hordei*, applying the chip-dPCR [27]. In a very recent study, Miles et al. performed the allele-specific detection of QoI fungicide-resistance in samples of grapevine powdery mildew using TaqMan probes in qPCR and ddPCR methods [28].

dPCR technologies enable the detection and analysis of nucleic acids at a level of sensitivity and precision beyond the capability of previous PCR-based methods. The availability of a ddPCR protocol, which can detect very low percentages of QoI resistance-conferring alleles, could be pivotal in order to detect the presence of resistant populations in field early on, offering a greater chance of adjusting spray timing, dosage, and mode-of-action usage by an evidence-driven resistance control strategy. To the best of our knowledge, in this study we present the development of the first ddPCR protocol for detecting and quantifying single nucleotide polymorphism, which confer fungicide resistance on *Z. tritici*. Using this approach, we investigated the presence and frequency of G143A substitution, which causes a reduction in *Z. tritici* sensitivity to QoIs, in cultures collected from fields of durum wheat that were treated with different fungicide spray programs.

## 2. Materials and Methods

### 2.1. Fungal Isolates

Three populations were collected in 2015 from three fields of *Triticum durum* located in Emilia-Romagna. Information about A, B, and C collected populations are summarized in Table 1.

To collect the populations, 20–25 leaves originating from different plants located in different positions in the field, were chosen from each field. All the detached leaves were placed in a humid chamber for 24 h, and at least 50 pycnidia were randomly transferred from the leaf upper side of different leaves to a single petri dish filled with PDA (Potato Destrose Agar, Becton, Dickinson & Company, Sparks, MD, USA).

Conidia suspensions were made from the plates of populations A and B, and were diluted at concentration of 10^5^ conidia/mL. The suspensions were spread onto PDA plates, and single conidia were transferred with a sterilized needle to new PDA plates. Each colony grown from single conidia was considered a monoconidial isolate. From populations A and B, 3 and 5 monoconidial isolates were obtained, respectively. The representative monoconidial cultures A.1 and B.1 were used to optimize ddPCR conditions and to test the sensitivity of the protocol, while populations A, B, and C were used as test samples for the ddPCR determination of the G143 and A143 allele frequencies.

### 2.2. DNA Extraction

Genomic DNA was purified by processing fresh mycelium scraped from PDA cultures. We extracted the populations’ gDNA by using Qiagen’s DNeasy Plant Mini Kit, while those of the monoconidial strains were obtained following a CTAB protocol [29]. All DNAs were tested for quality and concentration using a NanoQuant infinite M200PRO spectrophotometer (Tecan Trading AG, Männedorf, Switzerland) and a Thermofisher Qubit 3.0 fluorometer. Genomic DNA was either frozen at −20 °C for long-term storage or kept in refrigeration at around 4 °C to be used within a few days.

### 2.3. Sanger Sequencing

All of the A and B monoconidial isolates were investigated for the presence of G143A substitution. DNA was used in a polymerase chain reaction (PCR) with primers described in Torriani et al. (2009) [7]. PCR reactions were performed by Bio-Rad T100 Thermal Cycler in a total volume of 40 µL containing 100 ng of gDNA, 4 µL of 10X Ex Taq buffer, 0.2 mM of each dNTP, 0.2 μM of each primer, and 1 U TaKaRa Ex Taq (Takara Bio, Europe). Reactions were run in the following conditions: initial heating 96 °C for 2 min, followed by 35 cycles of 1min denaturation at 96 °C, 45 s annealing at 54.2 °C, 1 min elongation at 72 °C, and final extension step at 72 °C for 5 min. A fragment consisting of 652 bp was amplified for each gDNA. The PCR products were purified using a GEL/PCR Extraction & Purification Kit (Fisher Molecular Biology, Rome, Italy, then sequenced by Sanger sequencing using the same oligonucleotides employed for PCR amplification. Sanger sequencing was done by Eurofins Genomics (Ebersberg, Germany). In order to check for single nucleotide polymorphism (SNP), data obtained from Sanger sequencing were aligned by Sequencer 5.6.4 (Gene Codes Corporation, Ann Arbor, MI, USA) against the *cyt b* sequence belonging to the complete mitochondrial genome (EU090238) of *Z. tritici* strain IPO323.

### 2.4. ddPCR Reaction Setup and Equipment

ddPCR experiments were conducted following the essential points of the checklist for Digital MIQE Guidelines [30,31] according to Droplet Digital™ PCR Applications Guide 2014 indications. All ddPCR experiments were carried out using a combined Mutation Detection Assay (FAM+HEX) (Catalog #10049047) provided by Bio-Rad for the quantification of G143A substitution in *Z. tritici* (assay name: Zt_cytb, Assay ID: dMDS336305949). The assay design was performed by Bio-Rad’s proprietary computational algorithms using the online ddPCR Assay Design tool, available at https://www.bio-rad.com/digital-assays (last accessed on 18 December 2021) [32], selecting the option “Mutation Detection Assay”. This assay was specifically designed for ddPCR and is compatible with QX200. It was provided by the manufacturer in a single tube, including primers and probes targeting mutant or wild-type alleles mixed together. A143 allele (R, QoI resistance-conferring allele) and G143 allele (S, wild-type allele) were targeted by FAM and HEX, respectively, [33] and the probes were quenched with Iowa Black. The assay was designed to target nucleotide position 428 of the *cyt b* sequence from *Z. tritici* strain IPO323. The MIQE context entered at Bio-Rad’s web page for the design of the ddPCR Mutation Detection Assay was as follows: CTGATGATGGCAACCGCATTCTTAGGGTATGTATTACCTTATGGTCAAATGTCTTTATGAG[G/C]AGCAACAGTTATAACTAACTTATTGAGTGCAATACCTTGAGTTGGACAAGACATAGTTGAA. The length of the expected amplicon was 98 bp, and primer and probe sequences were designed by the tool as described in the primer sequence disclosure clarification of the MIQE Guidelines [34]. No details were provided by the vendor. The software returned only one assay option; this *in silico* validated assay had not been predesigned and/or wet-lab validated by Bio-Rad, so its performance needed to be validated prior to use.

For all experiments, a mix of 20 µL was prepared for each well using nuclease-free water. For all reactions, the components were used with final concentrations of the following: 1X Bio-Rad ddPCR^TM^ supermix for probes (no dUTP, 2X concentrated), 1X dd assay primers-probe mix (20X concentrated ready-to-use), 900 nM primers/250 nM for each probe. The gDNA amount added to the reactions is specified below in the section for each experiment. DNAs were not enzymatically digested prior to being used in ddPCR analysis. Two replications of no-template control (NTC) were tested during each ddPCR experiment of DNA amplification. Droplets were generated by a QX 200 Droplets Generator (Bio-Rad) in 8-well cartridges (Bio-Rad, cod. 186-4008). PCRs were performed by a Bio-Rad T100 Thermal Cycler. After the amplification, PCR products were transferred to a QX200 ddPCR reader (Bio-Rad) and absolute quantification experiments were performed. Data from the ddPCR reader were analyzed by Quanta Soft Version 1.7.4.0917. The threshold was manually set using a 2D-cluster plot, to allow the best identification of positive and negative partitions.

### 2.5. Evaluation of DNA Amplification by ddPCR Assay Using Standard PCR

For the amplification of DNA with primers contained in the Zt_cytb assay, standard PCR was carried out on gDNA purified from A.1 and B.1 monoconidial cultures. PCR reactions were performed by a Bio-Rad T100 Thermal Cycler in a total volume of 50 µL containing 50 ng of gDNA, 5 µL of 10X Ex Taq buffer, 0.2 mM of each dNTP, 0.2 μM of each primer, and 1.25 U TaKaRa Ex Taq (Takara Bio, Europe) with the following program: 95 °C for 3 min; 35 cycles of 95 °C for 30 s, 54 °C for 30 s, 72 °C for 20 s; then 10 min at 72 °C. A fragment consisting of 98 bp was expected to be obtained for each gDNA. PCR products were visualized on 1.2% agarose TAE gel stained with GelRed (Biotium, Inc. Fremont, CA, USA).

### 2.6. Preliminary Experiments of Droplet Generation

To ensure the production of a proper partition in our conditions, and in order to test the cleanliness of the environmental work conditions, two preliminary independent experiments of droplet generation were carried out, each by a different operator. Four replications were made by each operator per experiment. PCRs were performed as follows: 95 °C for 10 min followed by 39 cycles at 94 °C for 30 s with a ramp speed of 2 °C/s, 60 °C for 1 min, and 98 °C for 10 min. Reactions were performed without DNA, both in order to test the presence of contamination in our laboratory and to save genomic material.

### 2.7. Optimization of ddPCR Conditions

Two experiments were run to optimize the annealing/extension temperature, and different amounts of DNA purified from monoconidial isolates A.1 and B.1 were individually tested. The first experiment was run at 56 °C, 58 °C, and 60 °C annealing/extension temperatures, and 5 ng, 2 ng, and 0.5 ng of DNA were added in the ddPCR reaction. During the second experiment, the annealing/extension step was performed at 52 °C, 54 °C, and 56 °C. In this experiment 0.1 ng of DNA were tested. For both experiments, the thermal profile was as follows: 95 °C for 10 min followed by 39 cycles at 94 °C for 30 s with a ramp speed of 2 °C/s, at the temperatures described above for 1 min, and 98 °C for 10 min.

### 2.8. Test of the ddPCR Protocol Sensitivity

In order to define the accuracy and sensitivity of allele frequency determination reached by the Zt_cytb assay at optimized conditions, we performed an experiment mixing the DNA of monoconidial isolates A.1 (carrying A143 allele) and B.1 (carrying G143 allele). A total of 0.1 ng of gDNA of monoconidial isolates A.1 and B.1 were quantified by a fluorometer as described previously, and the copy number of G143 and A143 alleles was determined in each gDNA by a ddPCR analysis. Then, two groups of samples were made by mixing the proper quantities of DNA of monoconidial isolates A.1 and B.1. Samples of groups 1 and 2 were obtained by keeping the copy number of one allele constant while the copy number of the other allele was serially diluted four times, to obtain nine samples for each group. The samples were prepared as reported in Table 2. Statistical analysis was performed using Excel 2013 to calculate the linear correlation between copy numbers measured by ddPCR and expected copy numbers in DNA mixture samples.

Furthermore, the optimized protocol was tested for its ability to determine the G143 and A143 allele frequencies in populations A, B, and C by adding 0.1 ng of DNA to the PCR reaction.

For all the experiments described in this section, the thermal protocol was run as follows: 95 °C for 10 min, followed by 39 cycles at 94 °C for 30 s with a ramp speed of 2 °C/s, 54 °C for 1 min, and 98 °C for 10 min.

### 2.9. Amplicon Sequencing

In order to validate ddPCR results for population C, an amplicon sequencing experiment was carried out by Bio-Fab Research srl (Rome, Italy). The population’s gDNA was amplified by standard PCR, with primers containing the overhang adaptor designed by Illumina for NGS amplicon sequencing.

Forward primer:

5′-TCGTCGGCAGCGTCAGATGTGTATAAGAGACAGCATAATGAGAGATGTAAAC-3′.

Reverse primer:

5′-GTCTCGTGGGCTCGGAGATGTGTATAAGAGACAGCCTGATACACCTAAAG-3′.

PCR-clean up steps used AMPure XP beads to purify the product, before and after the attachment of Illumina indexes (Nextera XT Index Kit, Illumina, San Diego, CA, USA). Product size assessment and final library validation were made by a 2100 Bioanalyzer Desktop System (Agilent Technologies, Santa Clara, CA, USA). PCR product was pooled and sequenced on Illumina MiSeq, using a Reagent Kit v3 (600 cycles, run 2 × 300 PE). The raw sequences obtained were cleaned up, filtered in order to discard reads with average quality lower than 33 of phred score, and merged using Cutadapt [35] and Pear [36]. Amplicon clusters were produced and analyzed by Usearch [37] and Vsearch [38]. Percentages of *cyt b* G143 and *cyt b* A143 alleles were calculated using the 50 bp region around the mutation site, following the guidelines for MiSeq Reporter Generate FASTQ Workflow.

## 3. Results

### 3.1. Sanger Sequencing

The PCRs performed on all of the A and B monoconidial strains produced fragments of the expected length. The alignment of the data obtained by Sanger sequencing allowed us to determine the presence of the G143 allele in the five monoconidial strains B and the A143 allele in the three monoconidial strains A [15].

### 3.2. DNA Amplification by ddPCR Assay Using Standard PCR

The Zt_cytb assay used in the PCR reaction generated the amplification of the expected clear single fragment of 98 bp from gDNAs of monoconidial culture A and B, without non-specific amplification (Figure S1).

### 3.3. Droplet Generation and Optimization of PCR Conditions

**Figure 1:**
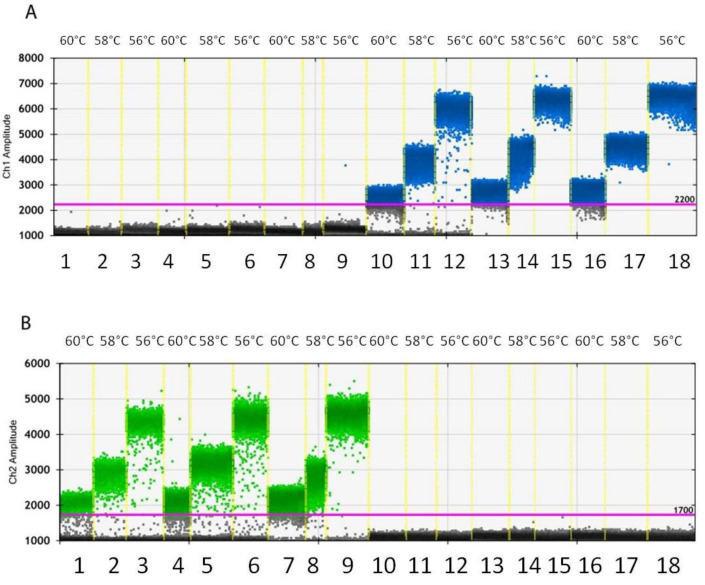
Amplitudes from channel 1 (FAM) (**A**) from channel 2 (HEX) (**B**) obtained during the first experiment to optimize the annealing/extension step and select the most performant DNA quantity. ddPCR assay was conducted at annealing/extension at 60 °C (lanes 1, 4, 7, 10, 13, 16), 58 °C (lanes 2, 5, 8, 11, 14, 17), or 56 °C (lanes 3, 6, 9, 12, 15, 18), and adding to the reaction 0.5 ng (lanes 1–3, 10–12), 2 ng (lanes 4–6, 13–15), or 5 ng (lanes 7–9, 16–18) of gDNA purified from the monoconidial isolate B (lanes 1–9) or from the monoconidial isolate A (lanes 10–18).

Overall, the results obtained from the preliminary experiments for droplet generation ranged from 11,978 to 17,169 total event number. No copies of G143 and A143 alleles were detected in the reactions, as expected, asserting that no contamination occurred, and a good technique was applied by the operators. In the first experiment aimed at optimizing a ddPCR protocol employing the Zt_cytb assay, the use of a higher quantity of DNA, such as 2 ng (Figure 1A, lanes 13–15) and 5 ng (Figure 1A, lanes 16–18), led to the saturation of the FAM signal at all of the annealing/extension tested temperatures. The signal saturation can be noted in these lanes because of the absence of the low-amplitude dark band representing the negative droplets, a condition which makes the application of Poisson statistics impossible. Reducing the DNA added to the reaction at 0.5 ng resulted in droplet separation in channel one (Figure 1A, lanes 10–12) and channel two (Figure 1B, lanes 1–3).

**Figure 2:**
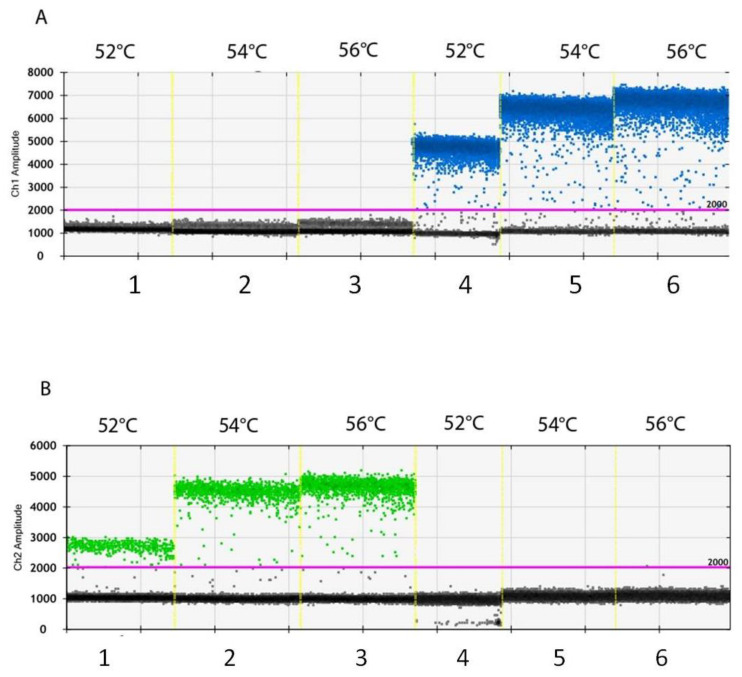
Amplitudes from channel 1 (FAM) (**A**) from channel 2 (HEX) (**B**) obtained during the second experiment to optimize the annealing/extension step and select the most performant DNA quantity. ddPCR assay was conducted for annealing/extension step, run at 52 °C (lanes 1, 4), 54 °C (lanes 2, 5), or 56 °C (lanes 3, 6) adding to the reaction 0.1 ng of gDNA purified from the monoconidial isolate B.1 (lanes 1–3) or from monoconidial isolate A.1 (lanes 4–6).

Even though the best tested annealing/extension temperature for both of the channels was identified as 56 °C, the results were not completely satisfactory. In a second experiment, lower annealing/extension temperatures were tested, and the DNA quantity was decreased to 0.1 ng (Figure 2). At these conditions, the clearest separation of positive and negative droplets occurred at 54 °C both in FAM (Figure 2A, lane five) and HEX (Figure 2B, lane two) channels. No copies of G143 and A143 alleles were detected in any of the NTCs (data not shown).

### 3.4. Sensitivity of ddPCR Optimized Protocol

**Figure 3:**
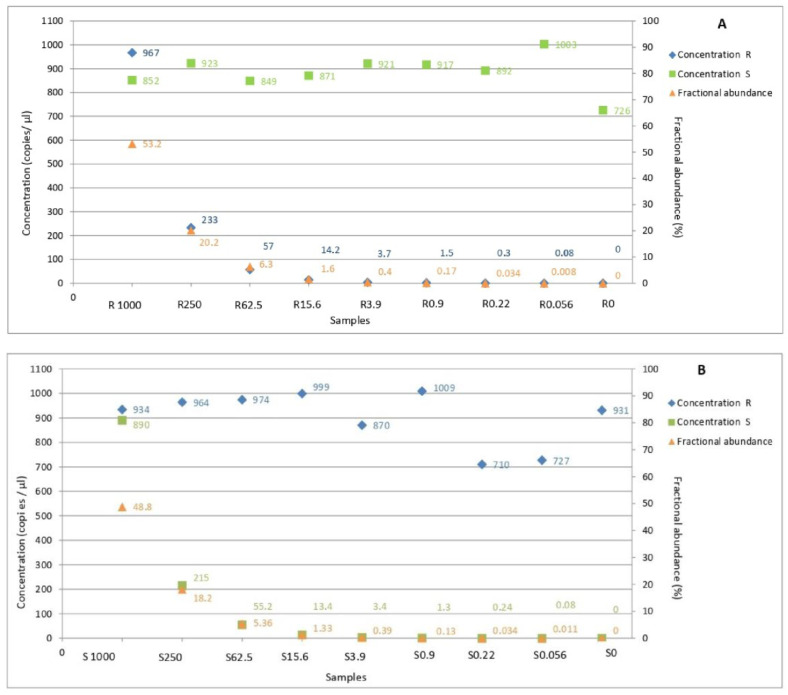
Values of concentration and fractional abundance of A143 and G143 alleles quantified in samples obtained by the mixture of gDNAs from A.1 and B.1 monoconidial isolates. A143 and G143 alleles are named in the graphic as R and S, respectively, and their copy numbers are represented by blue and green squares. (**A**) Samples from group 1: orange diamond represents the fractional abundances of A143 allele. (**B**) Samples from group 2: orange diamond represents the fractional abundances of G143 allele.

ddPCR was able to properly quantify the known copy number composition for all of the samples of group one and group two, as shown in Figure 3. The allele frequency was expressed as the percentage of fractional abundance as follows: [allele X/(allele X + allele Y)] 100. The lowest allele frequency determined by the assay applied at the optimized conditions on mixed samples was 0.008% for the resistant allele (Figure 3A) and 0.011% for the wild-type allele (Figure 3B). No copies of A143 and G143 alleles were detected in sample n.9 of group one (copies: 1000 S/0 R) nor in sample n.9 of group two (copies: 0 S/1000 R). No copies of either allele were detected in the two NTC replications (data not shown). Overall, the data allowed us to empirically determine the limit of the detection of the A143 allele concentration as being equal to 0.056 copies/μL.

**Figure 4:**
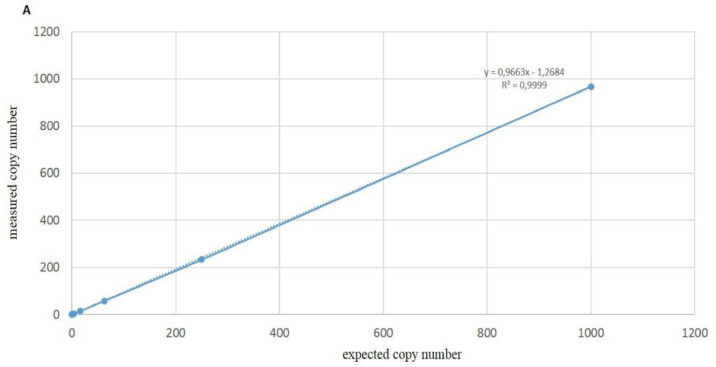

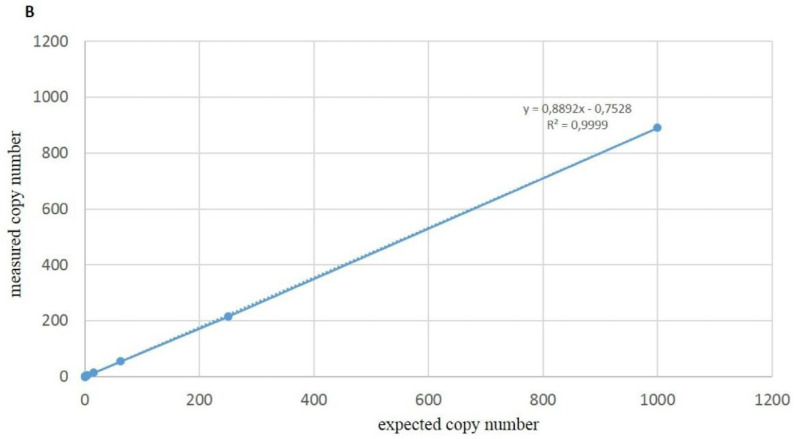
Linear correlation between values of measured copy number quantified by ddPCR (*y*-axis) and the known copy number composition of samples obtained by the mixture of gDNAs from A.1 and B.1 monoconidial isolates (*x*-axis). (**A**) Quantification of allele A143 in samples of group 1. (**B**) Quantification of allele G143 in DNA mixture of group 2.

The linear correlation between the real and the expected copy number of G143 (S) and A143 (R) alleles showed an R^2^ value of 0.9999 for both of them (Figure 4A,B).

### 3.5. Determination of Allele Frequency in Populations

**Figure 5:**
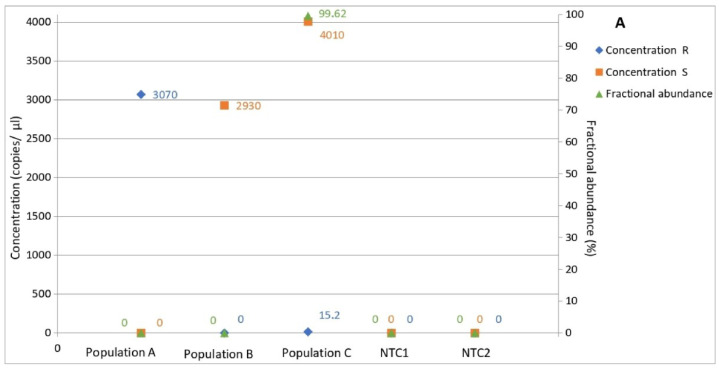

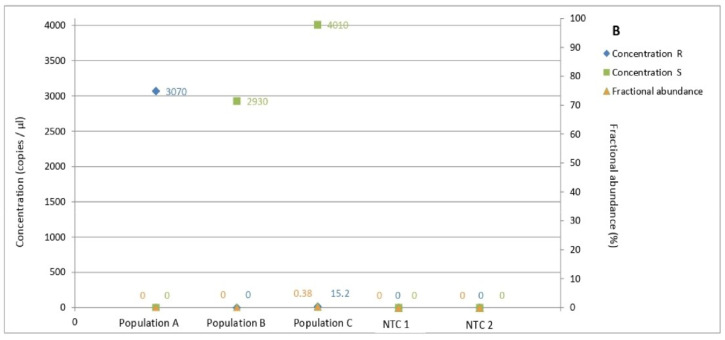
Determination of copy number and fractional abundance of A143 and G143 alleles in three field populations of *Z. tritici*. A143 and G143 alleles are named in the graphic as R and S, respectively, and their copy numbers are represented by blue and green squares. (**A**) Orange diamonds represent the fractional abundances of G143 allele. (**B**) Orange diamonds represent the fractional abundances of A143 allele. Results for no-template control replications are shown in NTC1 and NTC2.

The optimized protocol was tested by adding to the ddPCR reaction 0.1 ng of purified gDNA from the populations A, B, or C. The assay was able to determine the copy number and the frequency of each allele in all of the populations, and no copies of either allele were detected in the NTCs (Figure 5). The concentration and fractional abundance results confirmed for populations A and B the A143 and G143 genotype, respectively, mirroring those of the A.1 and B.1 monoconidial strains. Interestingly, population C exhibited 15.2 copies/µL of allele A143 and 4010 copies/µL of allele G143, resulting respectively in a frequency of 0.38% of the mutated allele (Figure 5B) and 99.62% of the wild-type allele (Figure 5A).

**Figure 6:**
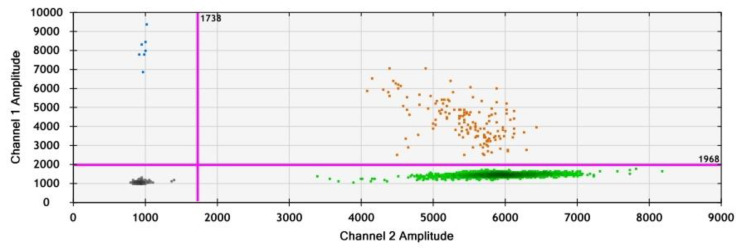
2D-cluster plot of droplet fluorescence for both targets in a mutation detection absolute quantification assay. FAM-positive (channel 1, A143 allele) droplets form the top-left blue cluster, HEX-positive (channel 2, G143 allele) droplets form the bottom-right green cluster, negative droplets for both targets form the bottom-left grey cluster, and positive droplets for both targets form the orange cluster.

When the output of the ddPCR analysis of population C was plotted on a 2D plot, each droplet sorted into one of four orthogonally arranged clusters, as expected for specific probes (Figure 6).

### 3.6. Determination of Allele Frequency in Population C by Amplicon Sequencing

The amplicon sequencing yielded 147,895 clean reads. Among the total number of accepted reads, G143 and A143 alleles were contained in 147,294 and 601 reads, respectively, consequently the calculated frequency of the wild-type allele was 99.59% while that of the allele conferring QoIs-resistance was 0.41%.

## 4. Discussion

Traditionally, to determine the frequency of alleles, a quantitative real-time PCR (qPCR) protocol is performed [39,40], but this method is time consuming because it requires the development of standard curves. Setting up ddPCR protocols is normally faster because calibration of the standard curve is not needed, also avoiding problems with reproducibility among and within laboratories.

Nevertheless, optimizing protocols continues to be an arduous task, which includes several steps for the ddPCR approach, as can be seen in the flowchart of Koch et al. (2016) [20]. Even though assays used for qPCR can be directly transferred to dPCR [23], as applied in the work by Miles et al. (2021) [28], whenever possible it is preferable to adopt an assay that fully address the criteria for the advised length and the other ideal conditions for primers and probes design, such as the targeting of mutant and wild-type alleles by FAM and HEX/VIC, respectively [41,42]. If the cost does not affect the budget, the best option could be a ddPCR Mutation Detection Assay that is wet-lab validated when available, or an in silico validated assay, such as those designed by Bio-Rad computational algorithms. These options made the protocol setup significantly safer due to several advantages, such as the higher confidence in the specificity of the signals of the probes, the reduction in the risk of probe cross-reactivity, and the avoidance of suboptimal TM assay design for ddPCR platform [42]. Moreover, the setup is faster because the optimal final concentrations of the reagents are already provided. The users should be able to order the Zt_cytb in silico validated assays using a commercial ID, however, high performances may be obtained from slightly different assays as long as they target the same region (identified by the MIQE context stated in Section 2.4) [34]. In the Zt_cytb assay, nucleotide position 428 of *cyt b* needs to be thought of as the “anchor” nucleotide.

In the ddPCR approach, the sensitivity of the measure is directly dependent on the droplet recovery that represents the number of software-accepted droplets per generated-droplet population [21]. Also, in order to allow for correct data processing, at least 10,000 droplet generation events are required [41]. More than 11,000 total events have been obtained by both operators during the preliminary experiments of droplet generation, and in all of the ddPCR experiments. These results could also depend on the use of cartridge 186-4008, which has been demonstrated to improve the number of partitions [22].

We used two different protocols to extract the DNA, including a CTAB method for the monoconidial isolates and a kit for the populations. Although it was demonstrated that the extraction method could influence the quality of the genomic DNA, affecting downstream analysis [24], in our case both approaches gave reliable ddPCR results. The fact that we obtained a single fragment of the expected size from standard PCR amplification performed using Zt_cytb assay clearly suggests that purified fungal gDNA are adequate for PCR-based analysis. With the aim of defining the sequence of the obtained 98-bp fragment using a commonly used method, such as Sanger sequencing, a cloning step would be required because of the shortness of the amplicon. This further investigation was deemed unnecessary because of the use of the dedicated Bio-Rad algorithm for the design of the Zt_cytb assay.

As advised for *in silico* validated assays, it was necessary to determine the best PCR conditions for the clear separation of positive and negative droplets, optimizing both the thermal protocol and the DNA quantity to be used in the reaction [20]. Only two experiments were required to identify the fully satisfactory amplification conditions. The saturation of the FAM channel, exhibited by 2 ng and 5 ng of DNA, bringing the A143 allele aligns with what was observed by Wang et al. (2018) [43]. The amplification of 0.5 ng of DNAs from monoconidial cultures A and B led to a compartment made up of droplets with intermediate fluorescence detected respectively in FAM and HEX channels. This compartment is called “rain” and was quickly resolved by assay condition optimization, as suggested by Rare Mutation Detection Best Practices Guidelines [42]. We identified 0.1 ng of DNA as the best performing DNA quantity. In this regard, we must remind readers that the target copy number could significantly differ in the 0.1 ng of DNA, depending on the quantification methods and genome degradation. It was observed that NanoDrop and Qubit overestimated the quantity of the genomic DNA, up to two times more, when compared with the ddPCR results [23]. Therefore, we advise adjusting the DNA quantity proposed in our protocol after a preliminary ddPCR run aimed at quantifying the copy number of the target in their samples.

In the study reported by Fraaije et al. (2005) [44], on G143A mutation presence in *Z. tritici* populations, qPCR determination by TaqMan probe-based assay of R allele frequency was 5–95% accurate. In the sensitivity-testing experiment, our optimized assay made it possible to achieve an accurate measurement of the allele copy numbers in all of the mixed samples of both of the groups, as suggested by the values of the linear correlation between the expected and measured values. The concentration of the allele copies quantified in sample n. 9 of group one and two represents the Zt_cytb assay LOD for A143 and G143 alleles, as indicated for its empirical estimation, as proposed by Rare Mutation Detection Best Practices Guidelines [42]. Similar results were obtained for the detection and quantification of mutations in the Cyp51 gene of *Blumeria graminis* f. sp. *hordei* using chip-dPCR technology [27].

Despite the presence of a high copy number for the other allele, it was possible to calculate accurately both A143 and G143 alleles’ frequencies to very low percentages (0.008% for R allele and 0.011% for S allele), when compared to many widely used methods for mutation analysis, which fail to detect sequences with abundances of less than one in 100 wild-type sequences [45,46,47].

The ddPCR assay revealed the presence of the genotype A143 and G143 in populations A and B respectively, and was able to detect and quantify the R allele at a very low frequency (0.38%, Figure 5B) as well as the S allele at a very high frequency (99.62%, Figure 5A) in population C. The orthogonality of clusters in the 2D plot shown in Figure 6 does not lead one to suspect probe cross-reactivity [20]. The results of percentages of G143 and A143 alleles in the natural population obtained from amplicon sequencing, performed by the external sequencing service, were compatible with allele frequencies calculated by ddPCR, confirming that ddPCR results were a real detection of low-level mutation. These results coincide with the origin of the samples (Table 1). In fact, population A was collected in an experimental field, therefore subjected to high fungicide pressure, and it was fully resistant in the biological assays. Population B, on the other hand, coming from an untreated field located far from other cultivated areas, showed full sensitivity [15]. Interestingly, population C, collected in a commercial field and never treated by QoIs during the year of sample collection, exhibited a low presence of reliably determined mutated allele. This result was probably justified by the history of the commercial site. Indeed, we do not know what treatments were applied in previous years, but it is likely that strobilurins fungicides were only slightly used.

Although currently it is still more expensive, the ddPCR approach can represent a valid alternative to qPCR. This study highlights the adequacy of digital PCR as an approach for fungicide resistance investigation, confirming what has already been proposed by Zulak et al. in 2018 [27], and extending it to the ddPCR method. In particular, the QX200 droplet-based dPCR platform is already widely used for routine mutation detection in clinics [48], making possible the creation of around 20,000 droplets together with a higher throughput (96-well plates are used) when compared with other dPCR methods [23]. Despite that fact that generating droplets for many samples takes a significant amount of time and requires expertise [28], in our opinion, it could be usefully employed for large monitoring studies, especially when early identification of resistance emergence can make a difference in the efficacy of the control strategy.

## 5. Conclusions

Our work proposes the first rapid, sensitive, and accurate ddPCR protocol that can measure the frequency of the alleles involved in *Z. tritici* fungicide resistance. The optimized protocol was successfully applied to investigate G143A substitution in Italian fungal strains collected in durum wheat fields during resistance monitoring, by analyzing gDNA that was purified from the fungal cultures of three populations and two monoconidial strains.

The availability of an approach that can detect such low percentages of resistant alleles may greatly help the setup of fungicide regimes that have the highest chances of controlling resistant strains and extending the life of effective fungicides.

This protocol is currently used in our laboratory for the analysis of a large set of Italian *Z. tritici* populations collected from durum and bread wheat. The tests on the DNAs of relevant wheat pathogens will be also performed, to extend the availability of the optimized Zy_cytb assay as a diagnostic tool for the same mutation in DNA directly extracted from STB-infected wheat leaves. Moreover, our ddPCR assays could be used in the future in validation of allele frequency results obtained by other analysis methods, such as the ASqPCR, as shown in a very recent paper published in 2021 by Dodhia [49], applying a digital PCR assay. Similar ddPCR assays could be developed in order to investigate other mutations involved in fungicide resistance in *Z. tritici* toward classes of fungicides other than QoIs, as well as in other plant pathogens.

## Figures and Tables

**Table 1:** Characteristics of *Z. tritici* populations investigated in this study.

Population	Site	Cultivar (Durum Wheat)	Fungicide Applications			
			Nr	Active Ingredients (Formulate)	Dose Rate/ha	Phenological Growth Stage
A	Dugliolo, Budrio (BO) (trial site)	San Carlo	from plots with 1 application	Pyraclostrobin + epoxiconazole (Opera New)	2 L	BBCH39
B	Sassoleone, Casalfiumanese (BO)	Miradoux	0	untreated	-	-
C	Medicina (BO)	San Carlo	3	tebuconazole + prochloraz (Orius P)	1.7 L	BBCH32
				bixafen + tebuconazole (Zantara)	1.5 L	BBCH39
				prochloraz + propiconazole (Novel Duo)	1.1 L	BBCH61

**Table 2:** Composition of mixed DNA samples. In group 1, nine samples were obtained by mixing 1000 copies of the G143 allele (S) with the serial dilution of copies of the A143 allele (R). Group 2 samples were composed to be the opposite of Group 1.

Groups	Copy Number of Alleles for Each Sample (S/R)								
	Sample 1	Sample 2	Sample 3	Sample 4	Sample 5	Sample 6	Sample 7	Sample 8	Sample 9
Group 1	1000/1000	1000/250	1000/62.5	1000/15.6	1000/3.9	1000/0.9	1000/0.22	1000/0.056	1000/0
Group 2	1000/1000	250/1000	62.5/1000	15.6/1000	3.9/1000	0.9/1000	0.22/1000	0.056/1000	0/1000

## Data Availability

Not applicable.

## Supplementary Materials

The following are available online at https://www.mdpi.com/article/10.3390/biology11020240/s1, Figure S1: Electrophoresis on agarose gel of fragments amplified by standard PCR from gDNAs of the monoconidial culture A.1 and B.1, using the Zt_cytb assay as reaction primers. A = monoconidal isolate A.1 (resistant), B = monoconidal isolate B.1 (sensitive), M1 = MassRuler DNA ladder (Thermo Fisher, USA), M2 =GeneRuler 100bp plus DNA ladder (Thermo Fisher), NTC = No Template Control.
