# Supplementary material for: A New Approach: Determining cyt b G143A Allele Frequency in Zymoseptoria tritici by Digital Droplet PCR
Source: Biology (Basel). 2022 Feb 4;11(2):240. doi: 10.3390/biology11020240 (PMC8869461; doi:10.3390/biology11020240)
Supplement: Supplementary file 1 [file biology-11-00240-s001.zip › biology-1541196-supplementary.pdf]

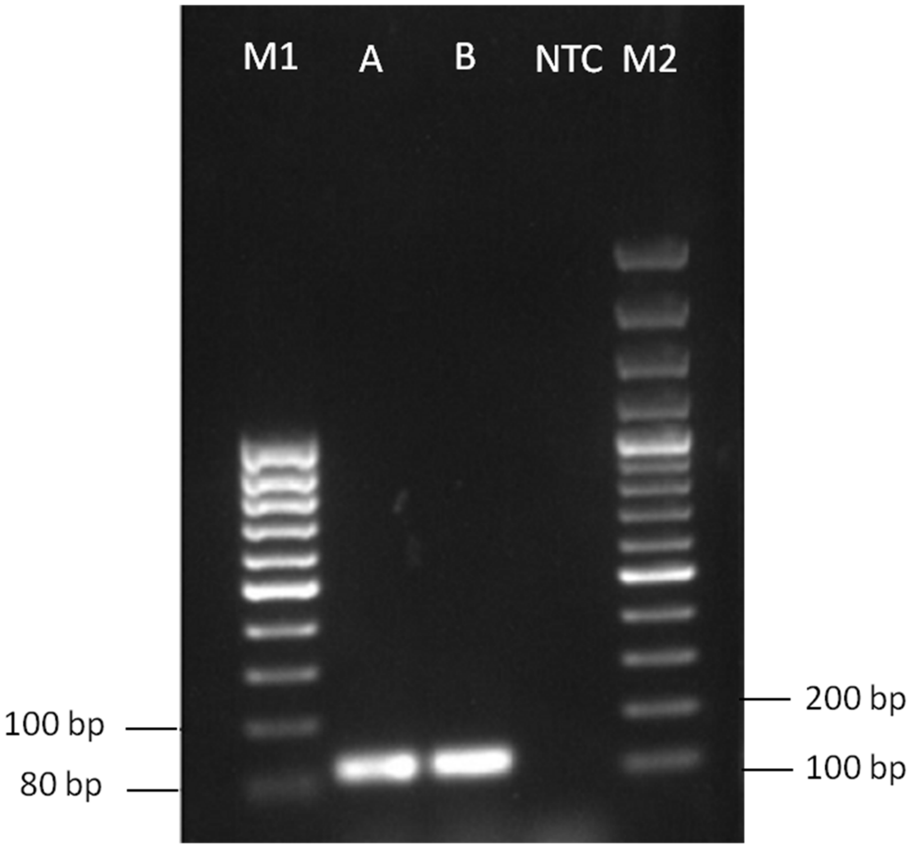

Figure S1. Electrophoresis on agarose gel of fragments amplified by standard PCR from gDNAs of the monoconidial culture A.1 and B.1, using the Zt_cytb assay as reaction primers. A = monoconidal isolate A.1 (resistant), B = monoconidal isolate B.1 (sensitive), M1= MassRuler DNA ladder (Thermo Fisher, USA), M2=GeneRuler 100bp plus DNA ladder (Thermo Fisher), NTC= No Template Control.
